# Two grass pollen tablets commercially available for allergy immunotherapy display different IgE epitope repertoires

**DOI:** 10.1186/s13601-019-0253-z

**Published:** 2019-02-27

**Authors:** Thierry Batard, Amparo Sanjuan, Laure Denis, Hélène Nguyen, Armelle Montagut, Joaquín Sastre, Sabina Rak, Jean F. Cuiné

**Affiliations:** 1 Product Development, Stallergenes Greer, 6 rue Alexis de Tocqueville, 92160 Antony, France; 2 Global Medical Affairs, Stallergenes Greer, 6 rue Alexis de Tocqueville, 92160 Antony, France; 3 Global Clinical Development, Stallergenes Greer, 6 rue Alexis de Tocqueville, 92160 Antony, France; 4 Fundación Jiménez Díaz, Madrid, Spain; 5 Sahlgrenska University Hospital, Gothenburg, Sweden

**Keywords:** Allergy immunotherapy, Blocking antibodies, Grass pollen, IgE epitope repertoire, Northern Europe, Patient sensitization profile, Southern Europe, Tablets

## Abstract

**Background:**

The distribution of Pooideae species varies across Europe. Especially, Timothy is less represented in Southern than in Northern Europe. Since allergenic cross-reactivity between pollens from different grasses is only partial, grass pollen-allergic patients are expected to display different sensitization profiles, with specific IgE directed against different combinations of allergenic epitopes, depending on their living places in Europe and the grasses they are exposed to. In this context, this study aimed at comparing two tablets commercially available for allergy immunotherapy, namely a 5-grass (Cocksfoot, Meadow-grass, Rye-grass, Sweet vernal-grass and Timothy) and a 1-grass (Timothy) pollen tablets, for their ability to represent the sensitization profiles of patients, depending on whether they live in Southern or Northern Europe.

**Methods:**

Sera were collected from adult patients living in Spain (n = 19) and Sweden (n = 22). Tablets were compared for their ability to inhibit the binding of patient serum IgE to pollen allergens from twelve grasses commonly distributed throughout Europe, as determined by the areas under the curves obtained by ELISA-inhibition. Tablets were adjusted to an equivalent allergenic activity, based on the CBER/FDA bioequivalent allergy unit.

**Results:**

Inhibition of the IgE binding to pollen allergens from twelve grasses was significantly stronger with the 5-grass than with the 1-grass pollen tablet (*p* < 0.0001), regardless of whether patients were considered as a whole or by geographical area. This difference between tablets was significantly greater for Southern than Northern European patients (*p* < 0.05).

**Conclusions:**

Compared to the 1-grass tablet, the 5-grass tablet generally covers better the sensitization profiles of European patients, especially patients from Southern Europe, in principle less exposed to pollen from Timothy than from other grasses. The 5-grass tablet is therefore expected to elicit larger spectra of blocking antibodies, which might have implications in light of the generally accepted mechanisms of allergy immunotherapy.

**Electronic supplementary material:**

The online version of this article (10.1186/s13601-019-0253-z) contains supplementary material, which is available to authorized users.

## Background

Grasses are estimated to cover a quarter of Earth’s land area [1]. Pollens emitted by grasses of the Pooideae subfamily are, with house dust mites, among the two most important sources of airborne allergens and causes of IgE-mediated allergies, especially in Europe [2].

The distribution of Pooideae species across Europe is known to vary between regions [3]. For instance, grass species such as Timothy (*Phleum pratense*) and Sweet vernal-grass (*Anthoxanthum odoratum*) are less represented in Southern Europe than in Northern Europe. As a consequence, exposure of grass pollen-allergic patients to various species will vary from one country to another. Although pollen allergens from the different Pooideae species display a high allergenic cross-reactivity, the latter is only partial, as shown with group 1 and group 5 allergens displaying species-restricted (or semi-restricted) IgE epitopes [4]. European grass-pollen allergic patients are therefore expected to display different IgE sensitization profiles, with IgE directed against different combinations of allergenic epitopes, according to their geographic location and the grass species they are exposed to. This may have important implications for allergy immunotherapy (AIT), based on a recent study from Kinaciyan et al. comparing the clinical efficacy on birch pollen-related apple allergy of two sublingual immunotherapy (SLIT) formulations containing either the apple allergen Mal d 1 or the birch pollen allergen Bet v 1, as recombinant molecules [5]. In that study, SLIT product containing rMal d 1 was significantly more efficient than SLIT product containing rBet v 1 in reducing Mal d 1-induced symptoms and treating birch pollen-associated apple allergy, despite IgE cross-reactivity between the two allergens resulting from their high structural homology [6–9]. This superiority in clinical efficacy could be attributed, at least in part, to the fact that Bet v 1 does not display all the possible IgE epitopes of Mal d 1, as a result of structural differences between the two allergens [8–10]. As such, Bet v 1 is expected to elicit a limited spectrum of blocking antibodies competing for Mal d 1 IgE epitopes, as compared to Mal d 1 itself. Given the reported role of such blocking antibodies in the clinical efficacy of AIT (reviewed in [11–14]), this might explain the lower efficacy of Bet v 1 in AIT treatment of Mal d 1-induced symptoms. The contribution of species-specific IgE epitopes in this treatment is even more likely, given that the contribution of species-specific T cell epitopes is probably modest. Indeed, amino acid substitutions in Mal d 1 T cell epitope sequences has a weak influence on T cell recognition, while it has a marked negative impact on IgE binding [15, 16]. Altogether, those data suggest that the clinical efficacy of an AIT product may be improved when it reflects the full repertoire of epitopes recognized by patients’ IgE, and is prone to elicit a larger spectrum of blocking antibodies. When the IgE epitope repertoire is expected to vary significantly depending on geographical areas, as with grass pollen allergens, the ability of an AIT product to cover this repertoire should be evaluated for corresponding patients’ subpopulations.

Today, two grass pollen tablets are commercially available for SLIT, namely ORALAIR^®^, containing a pollen extract of 5 grasses (Cocksfoot, Meadow-grass, Rye-grass, Sweet vernal-grass and Timothy), and Grazax™/Grastek^®^, containing a single grass (Timothy) pollen extract. The present study aimed at comparing the ability of the two tablets to mimic the repertoires of IgE epitopes recognized by grass pollen-allergic patients from different European geographies (namely, Northern versus Southern Europe).

## Methods

### Sera

Blood was obtained from grass-pollen allergic adult patients living either in Northern Europe (Sweden, namely in the vicinity of Gothenberg), or in Southern Europe (Spain, in the vicinity of Madrid).

Inclusion criteria were female or male outpatients 18 years of age or more, with known symptoms of grass pollen allergy for at least two consecutive seasons, sensitized to at least one identified grass pollen as evidenced by positive skin prick test and/or specific serum IgE ≥ 10 kU/L and who have been living at least 10 years in the corresponding study country.

Exclusion criteria were: pregnancy, extensive skin disease likely to jeopardize the skin testing, AIT to grass pollen within the last 7 years, autoimmune diseases, immune complex or immune deficiency diseases, previous or concomitant medication that impairs immune response or suppresses the immediate skin test response, human immunodeficiency virus type 1 or 2 infection, hepatitis B or C virus infection, systemic disease interfering with the study conduct or outcome, alcohol dependence, and current participation in an interventional clinical trial.

A total of 20 Spanish subjects, numbered from 00101 to 00120, were included in the study and drawn. They were 22 to 54 years old, 9 of them (45%) being females. Since Spanish patient 00105, a 33 years old male, turned out to be erroneously considered skin prick test positive and had no grass pollen-specific IgE test performed, it was excluded from the per protocol set.

Also, a total of 23 Swedish patients, numbered from 00201 to 00223, were included in the study and drawn. They were from 19 to 52 years old, 11 of them (48%) being females. Because of insufficient amount of blood drawn, Swedish patient number 00204, a 48 years old male, did not have any in vitro testing and was excluded from the full analysis set (see Additional file 1: Tables S1 to S7 for more details on Spanish and Swedish patients).

After blood sampling, patient sera were obtained by centrifugation, immediately aliquoted and frozen at − 20 °C until analysis.

### Grass pollen extracts and tablets

A pollen extract of 12 grasses commonly present in Europe (Bent grass, Bermuda grass, Brome grass, Cocksfoot, False oat-grass, Meadow fescue, Meadow-grass, Rye-grass, Sweet vernal-grass, Timothy, Wild oat and Yorkshire fog) was coated onto 96-well microtiter enzyme-linked immunosorbent assay (ELISA) plates (Costar CLS 3590-100EA, Sigma-Aldrich, France). Additional file 1: Figure S1 displays the electrophoretic profile of this 12-grass pollen extract. This extract was obtained by a 5% mass/volume (m/v) extraction of pollens in 50 mM ammonium bicarbonate for 24 h at + 5 °C under stirring conditions, concentrated and freeze-dried with 2% (v/v) mannitol. The extract was reconstituted to obtain an allergenic potency of 100 IR/ml [17] and diluted 20 folds in phosphate-buffered saline (PBS) for coating.

In a set of control experiments, a 1-grass Timothy pollen extract, obtained following the same procedure as for the 12-grass pollen extract, was reconstituted to obtain an allergenic potency of 100 IR/ml and diluted 20 folds in PBS for coating.

Two commercially available grass pollen tablets were evaluated, namely ORALAIR^®^ 300 IR (batch No. 3132-1, expiration date: November 2017; Stallergenes Greer, Antony, France), a tablet of pollen extract from 5 grasses (Cocksfoot, Meadow-grass, Rye-grass, Sweet vernal-grass and Timothy), and Grazax™/Grastek^®^ 75,000 SQ-T (batch No. R1159, expiration date: January 2020; ALK-Abelló, Hørsholm, Denmark), a tablet of Timothy pollen extract, hereafter referred to as 5-grass pollen tablet and 1-grass pollen tablet, respectively. Importantly, the recommended dose is the same for both ORALAIR and Grazax, namely one tablet per day.

### ELISA-inhibition

After reconstitution and dilution in PBS, 1-grass or 12-grass pollen extracts were coated onto 96-well polystyrene microtiter ELISA plates (Costar CLS 3590-100EA Sigma-Aldrich, France), and incubated overnight at + 5 °C. After washing with PBS-Tween (PBS containing 0.1% (v/v) Tween 20), the microtiter wells were saturated with 1% (m/v) bovine serum albumin in PBS during 3 h at room temperature, and washed again before competition assay.

For ELISA-inhibition, one 5-grass pollen tablet and three 1-grass pollen tablets were reconstituted in 1 ml of dilution buffer (PBS containing 0.05% (v/v) Brij 35 and 0.1% (m/v) bovine serum albumin) to obtain an equivalent potency, based on bioequivalent allergy unit (BAU) [18]. Reconstituted tablets were mixed by rotative stirring at + 5 °C for 5 min and centrifuged for 15 min at 1000*g* and + 5 °C. The supernatants were recovered, homogenized by vortexing and submitted to serial dilutions in dilution buffer. Each dilution was co-incubated in duplicate with the patient serum to be tested, and allowed to compete with coated allergens for the binding of the specific IgE from patient serum for 2 h at room temperature. For a given patient serum, 5-grass and 1-grass pollen tablets were compared in ELISA-inhibition using a same microtiter plate.

After washing, the IgE that remained bound onto the microtiter well-coated allergens were detected using goat IgG anti-human IgE conjugated with horseradish peroxidase (Eurobio, Courtabœuf, France). After a 2-h incubation at room temperature, the plates were washed again and tetramethylbenzidine/H_2_O_2_ chromogenic substrate (Eurobio, Courtabœuf, France) was added into the wells. The reaction was stopped with 1 M phosphoric acid. Absorbances were read using a dual wavelength setting of 450 nm and 620 nm on a Versa Max spectrophotometer (Molecular Devices, Evry, France) with SoftMaxProGxP software version 5.4.1. The area under the curve (AUC) was then calculated for each inhibition curve obtained, using the trapezoidal rule.

To confirm potency equivalence between one 5-grass pollen tablet and three 1-grass pollen tablets, control experiments were performed exactly as described above, except that ELISA plates were coated with a 1-grass Timothy pollen extract, instead of a 12-grass pollen extract. Due to insufficient amount of serum, Spanish patients 00103 and Swedish patients 00201 and 00203 could not be evaluated in those control experiments.

### Statistics

AUC and exact two-sided Wilcoxon signed-rank and rank-sum tests were computed using SAS^®^ for Windows, version 9.4 (SAS Institute, Cary, North Carolina, USA).

## Results and discussion

In the current study, two grass SLIT tablets of different compositions, the 5-grass pollen tablet ORALAIR^®^ and the 1-grass pollen tablet Grazax^®^/Grastek^®^, were compared with regard to their ability to mimic the various repertoires of epitopes recognized by the IgE from grass pollen-allergic European patients. Common Pooideae species being differently distributed across Europe, patients from Spain and Sweden were selected to reflect the differences observed between Nothern and Southern Europe [3].

To compare the ability of the two grass tablets to mimic the repertoires of epitopes recognized by grass pollen-allergic patients IgE, the two tablets were evaluated at equivalent allergenic activity, as expressed in the CBER/FDA common BAU unit for grass pollen extracts [18]. This allowed to avoid a bias due to different biological potencies. More precisely, this normalization prevents an observed difference between the two products from being due to different concentrations of allergens rather than to a different number of epitopes displayed by the allergens originating from those different products. To this end, a single 5-grass pollen tablet (9000 BAU) was compared to three 1-grass pollen tablets (2800 BAU per tablet). Inhibition experiments with ELISA microtiter plates coated with a Timothy pollen extract, such as those performed for BAU measurement [18], confirmed that three 1-grass pollen tablets display an allergenic activity comparable to that of one 5-grass pollen tablet, since no significant difference was observed between the areas under the corresponding inhibition curves (AUCs), as exemplified in Additional file 1: Fig. S2 and reported in Additional file 1: Figs. S3 to S5. In contrast, a single 1-grass pollen tablet displays a significantly lower allergenic activity than one 5-grass pollen tablet (Additional file 1: Figs. S6 to S8), providing a statistical confirmation of already published data [18].

**Fig. 1:**
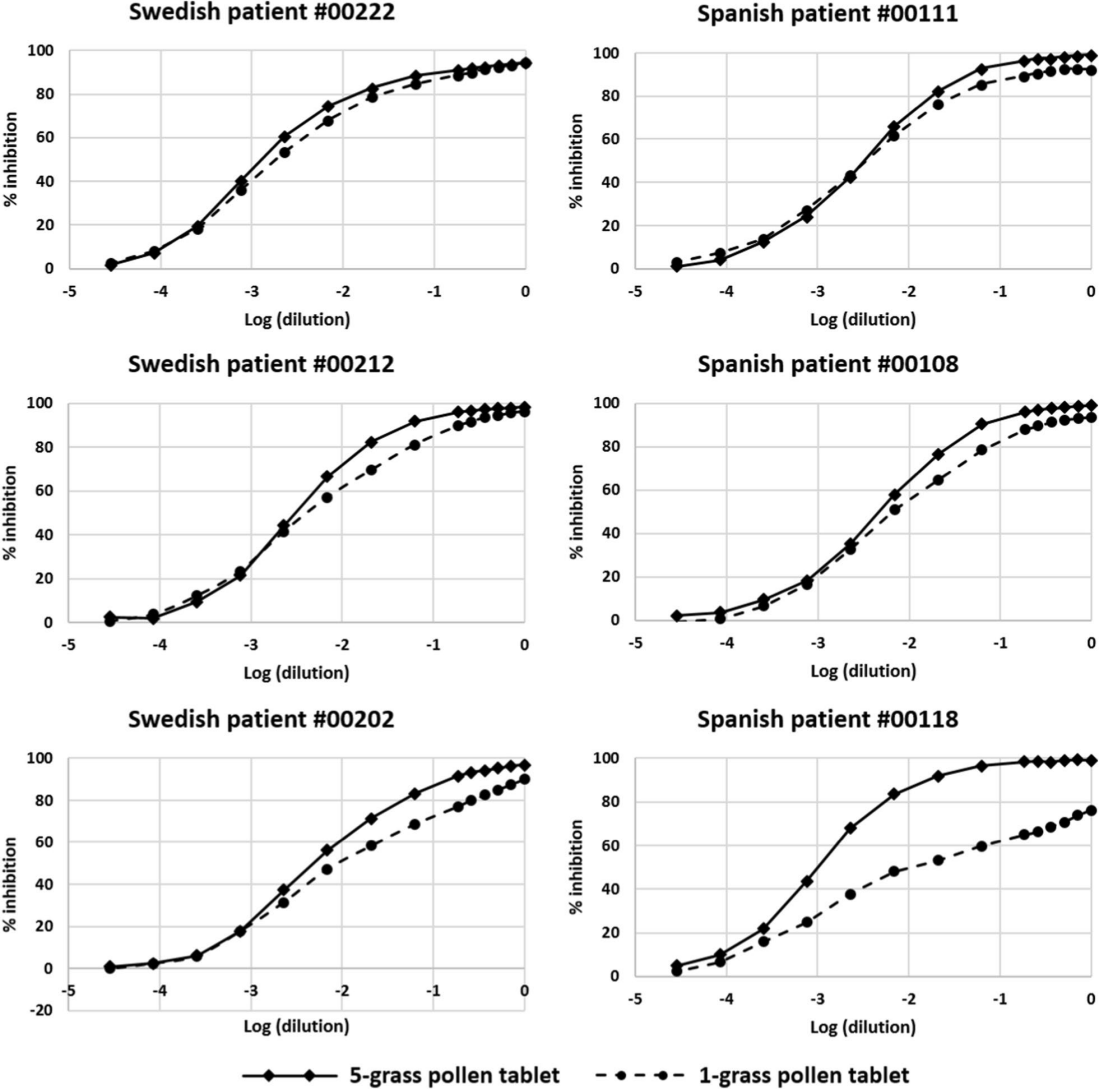
Inhibition curves corresponding to one tablet of 5-grass pollen extract (plain lines and losanges) and three tablets of 1-grass pollen extract (dashed lines and plain circles). Increasing dilutions of tablets were allowed to compete with immobilized 12-grass pollen allergens for the binding of serum IgE from a grass pollen-allergic patient, and the IgE that remained bound to the immobilized allergens were detected. Patients were classified in 3 tertiles, according to the difference between the AUC obtained with the 5-grass pollen tablet and the one obtained with three 1-grass pollen tablets. Patients of the first, second and third tertiles are exemplified, respectively, by Swedish patient #00222 and Spanish patient #00111 (top), Swedish patient #00212 and Spanish patient #00108 (middle), and Swedish patient #00202 and Spanish patient #00118 (bottom)

**Fig. 2:**
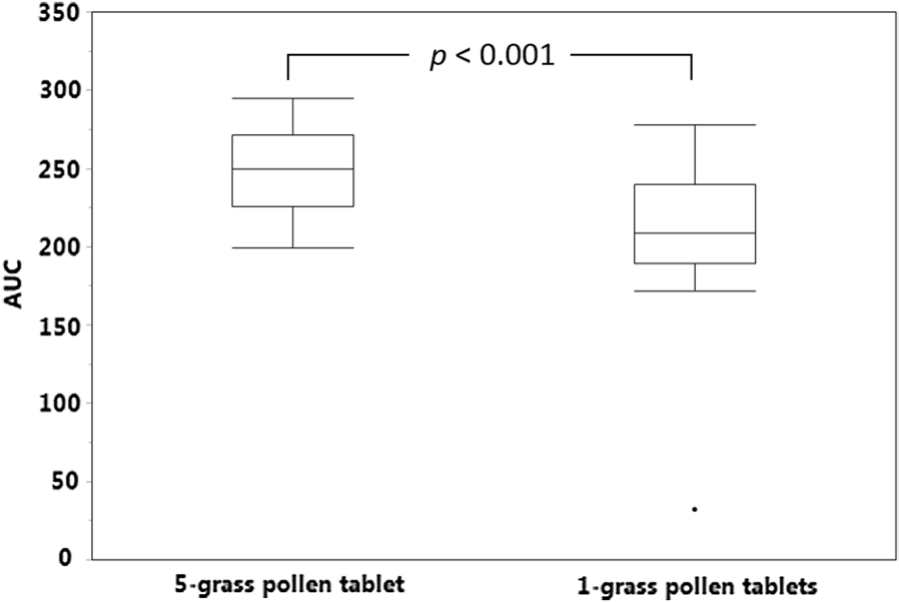
AUCs obtained with one 5-grass pollen tablet and three 1-grass pollen tablets. AUCs were obtained in ELISA-inhibition experiments using microtiter plates coated with a 12-grass pollen extract and detection of serum IgE from Spanish patients (*n* = 19). The *p*-value was obtained with the two-sided Wilcoxon signed rank test

**Fig. 3:**
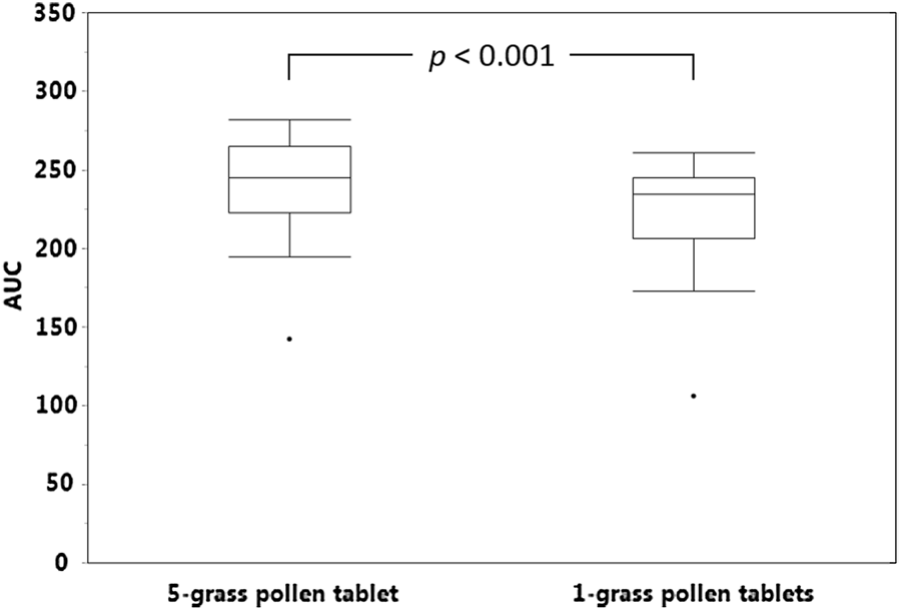
AUCs obtained with one 5-grass pollen tablet and three 1-grass pollen tablets. AUCs were obtained in ELISA-inhibition experiments using microtiter plates coated with a 12-grass pollen extract and detection of serum IgE from Swedish patients (*n *= 22). The *p*-value was obtained with the two-sided Wilcoxon signed rank test

**Fig. 4:**
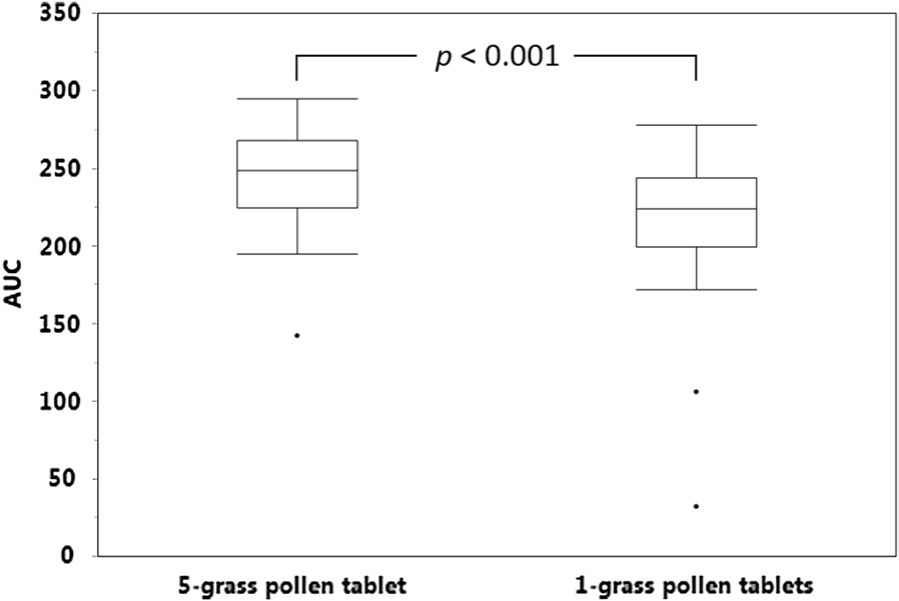
AUCs obtained with one 5-grass pollen tablet and three 1-grass pollen tablets. AUCs were obtained in ELISA-inhibition experiments using microtiter plates coated with a 12-grass pollen extract and detection of serum IgE from both Spanish and Swedish patients (*n *= 41). The *p*-value was obtained with the two-sided Wilcoxon signed rank test

**Fig. 5:**
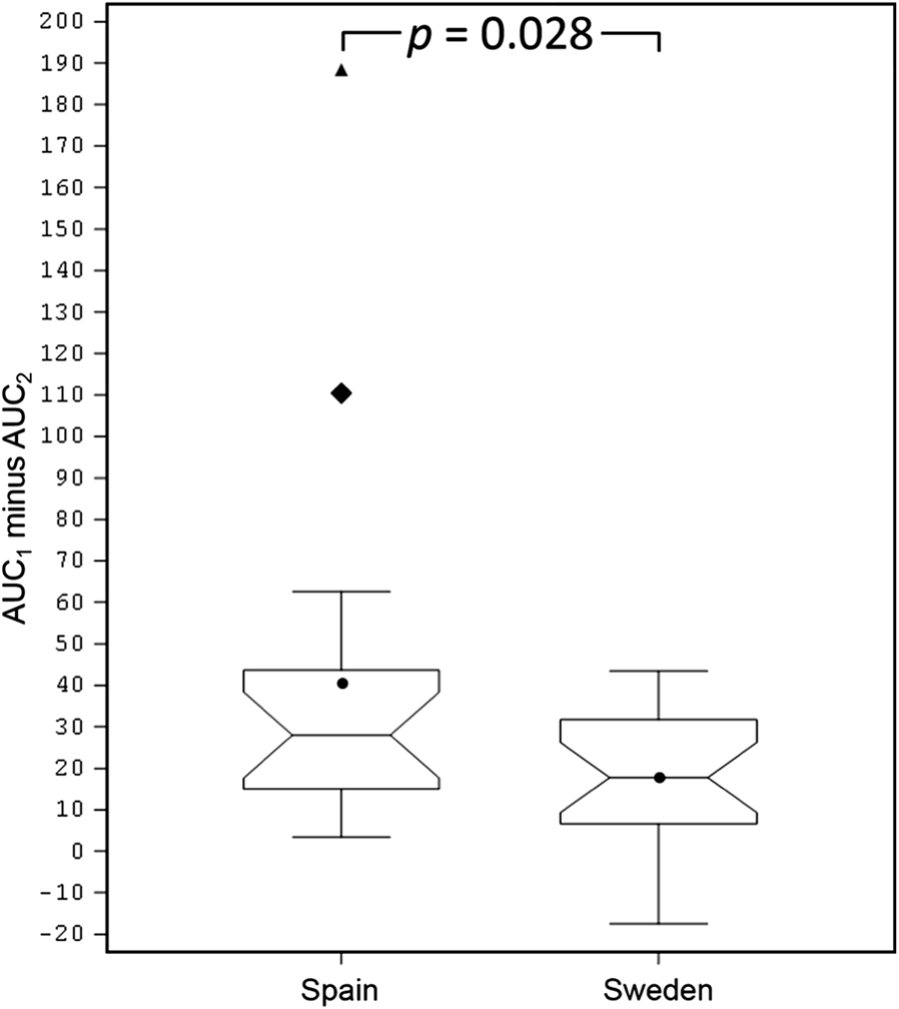
AUC_1_ minus AUC_2_ by country. AUC_1_ is the area under the inhibition curve obtained after competition of increasing dilutions of one 5-grass tablet for the binding of serum IgE from Spanish and Swedish patients (*n *= 19 and 22, respectively) to immobilized allergens of a 12-grass pollen extract, and detection of the IgE that remained bound to the immobilized allergens; AUC_2_ is the area under the inhibition curve obtained in the same conditions with increasing dilutions of three 1-grass pollen tablets. The triangle and losange are, respectively, a severe outlier and an outlier corresponding to Spanish patients #00103 and #00118. The *p*-value was obtained with the exact two-sided Wilcoxon rank-sum test

Subsequently, the ability of the two products to mimic the IgE epitope repertoire of the pollens from twelve grasses commonly present in the environment across Europe was evaluated, the twelve grasses being chosen so as to reflect as far as possible the extent of pollen exposure of European patients to the different grass species [3]. To this end, one 5-grass pollen tablet and three 1-grass pollen tablets were allowed to compete in ELISA-inhibition experiments with a 12-grass pollen extract for the binding of serum IgE from the study patients. The whole set of patients was stratified in 3 tertiles according to the difference between the AUCs obtained with the two different tablets. As exemplified in Fig. 1 for each tertile and each population, the 5-grass pollen tablet displayed on average a higher capacity to compete with pollen allergens from the twelve grasses for the binding of serum IgE. This difference was statistically highly significant (*p *< 0.001), whether patients were analyzed per country of residence or as a whole population (Figs. 2, 3, 4). Those significant differences indicate that the 5-grass pollen tablet contains IgE epitopes that are present in pollens of the 12 grasses but not in the 1-grass pollen tablet. In other words, compared to the 1-grass pollen tablet, the 5-grass pollen tablet shows a higher ability to mimic the repertoire of IgE epitopes of pollens from twelve grasses commonly present in the environment across Europe. This is especially true when those epitopes are the ones recognized by IgE from Southern European patients, as expected from their comparatively lower exposition to Timothy pollen [3]. Indeed, the difference observed between the 5-grass and 1-grass pollen tablets was significantly greater (*p* < 0.03) when evidenced by using sera from Spanish patients, as compared to sera from Swedish patients (Fig. 5). This reflects a differential sensitization profile of patients from Southern to Northern Europe, mirroring the distribution of grass species in these European geographies [3].

The fact that a larger IgE epitope repertoire was evidenced for the 5-grass tablet, as compared to the 1-grass pollen tablet, does not mean that the former contains groups of allergens that are not present in the latter. Indeed, almost all—if not all—groups of allergens present in the pollens used to manufacture the 5-grass pollen tablet are present in the Timothy pollen used to manufacture the 1-grass pollen tablet [19–21]. Therefore, it is most likely that the differences observed between the two tablets is rather due to species-specific epitopes that are present on allergens from the 5-grass pollen tablets, e.g. Dac g 1, Lol p 1, Poa p 1 and Ant o 1, but not on the homologous allergens present in the 1-grass pollen tablet, e.g. Phl p 1. In this respect, a Timothy pollen extract was shown to poorly inhibit the binding of IgE from some patients to Dac g 1, Lol p 1, Poa p 1 and Ant o 1 (from Cocksfoot, Rye-grass, Meadow-grass and Sweet vernal-grass pollens, respectively), while it almost totally inhibited the binding of IgE to the Timothy pollen Phl p 1 allergen, as expected [4].

Such a difference between the 5-grass and 1-grass pollen tablets in terms of IgE epitope repertoire might have important implications in patients treated by AIT. Indeed, blocking antibodies—especially of the IgG4 subclass—to IgE epitopes have been extensively described as potential contributors to the efficacy of AIT (reviewed in [11–14]). On this basis, AIT products should encompass as much as possible the different repertoires of IgE epitopes recognized by the patient population or subpopulation to be treated, so as to elicit the most complete spectra of blocking antibodies. To this end, AIT products should not simply rely upon allergenic cross-reactivity between species involved in sensitization, but take into account the epitope specificity of the different species, hence a previous recommendation to manufacture grass pollen AIT products with pollens from grass species found in various geographical areas [22].

Covering a large spectrum of IgE epitopes in grass pollen AIT products might become even more critical for Northern European patients in the near future, due to shifting of some grass species to the North, or to progressive decline of other grasses in this geography, as a consequence of global warming [23, 24].

## Conclusions

The repertoires of grass pollen allergenic epitopes recognized by the IgE from European patients are generally underrepresented in the 1-grass pollen Grazax™/Grastek^®^ tablet when compared to the 5-grass pollen ORALAIR^®^ tablet. The underrepresentation of the IgE epitope repertoires in the 1-grass pollen tablet is even more pronounced for patients living in Southern Europe, when compared to those living in Northern Europe. In light of the generally accepted mechanisms of AIT, this might have important implications.

## Additional file

**Additional file 1.** Additional file figures and tables.

## Abbreviations

AIT: allergy immunotherapy
AUC: area under the curve
BAU: bioequivalent allergy unit
CBER: Center for Biologics Evaluation and Research
ELISA: enzyme-linked immunosorbent assay
FDA: Food and Drug Administration
m: mass
PBS: phosphate-buffered saline
SLIT: sublingual immunotherapy
v: volume
