# Supplementary material for: Two grass pollen tablets commercially available for allergy immunotherapy display different IgE epitope repertoires
Source: Clin Transl Allergy. 2019 Feb 27;9:13. doi: 10.1186/s13601-019-0253-z (PMC6391756; doi:10.1186/s13601-019-0253-z)
Supplement: Supplementary file 1 — Additional file 1. Additional file figures and tables. [file 13601_2019_253_MOESM1_ESM.docx]

**Additional file figure legends**

**Additional file 1: Fig. S1 Electrophoretic profile of the 12-grass pollen extract used for coating the microtiter ELISA plates.** The profile was obtained by SDS-PAGE followed by SYPRO Ruby staining. m(kDa) stands for molecular mass (as expressed in kilodaltons).

**Additional file 1: Fig. S2. Inhibition curves corresponding to one tablet of 5-grass pollen extract (plain line and losanges) and three tablets of 1-grass pollen extract (dashed line and plain circles).** Increasing dilutions of tablets were allowed to compete with immobilized 1-grass (Timothy) pollen allergens for the binding of serum IgE from Spanish pollen-allergic patient 00118.

**Additional file 1: Fig. S3. AUCs obtained with one 5-grass pollen tablet and three 1-grass pollen tablets.** AUCs were obtained in ELISA-inhibition experiments using microtiter plates coated with a 1-grass Timothy pollen extract and detection of serum IgE from Spanish patients (*n* = 18). The *p*-value was obtained with the two-sided Wilcoxon signed rank test.

**Additional file 1: Fig. S4. AUCs obtained with one 5-grass pollen tablet and three 1-grass pollen tablets.** AUCs were obtained in ELISA-inhibition experiments using microtiter plates coated with a 1-grass Timothy pollen extract and detection of serum IgE from Swedish patients (*n* = 20). The *p*-value was obtained with the two-sided Wilcoxon signed rank test.

**Additional file 1: Fig. S5. AUCs obtained with one 5-grass pollen tablet and three 1-grass pollen tablets.** AUCs were obtained in ELISA-inhibition experiments using microtiter plates coated with a 1-grass Timothy pollen extract and detection of serum IgE from both Spanish and Swedish patients (*n* = 38). The *p*-value was obtained with the two-sided Wilcoxon signed rank test.

**Additional file 1: Fig. S6. AUCs obtained with one 5-grass pollen tablet and one 1-grass pollen tablets.** AUCs were obtained in ELISA-inhibition experiments using microtiter plates coated with a 1-grass Timothy pollen extract and detection of serum IgE from Spanish patients (*n* = 18). The *p*-value was obtained with the two-sided Wilcoxon signed rank test.

**Additional file 1: Fig. S7. AUCs obtained with one 5-grass pollen tablet and one 1-grass pollen tablets.** AUCs were obtained in ELISA-inhibition experiments using microtiter plates coated with a 1-grass Timothy pollen extract and detection of serum IgE from Swedish patients (*n* = 20). The *p*-value was obtained with the two-sided Wilcoxon signed rank test.

**Additional file 1: Fig. S8. AUCs obtained with one 5-grass pollen tablet and one 1-grass pollen tablets.** AUCs were obtained in ELISA-inhibition experiments using microtiter plates coated with a 1-grass Timothy pollen extract and detection of serum IgE from both Spanish and Swedish patients (*n* = 38). The *p*-value was obtained with the two-sided Wilcoxon signed rank test.

**Additional file 1: Fig. S1**


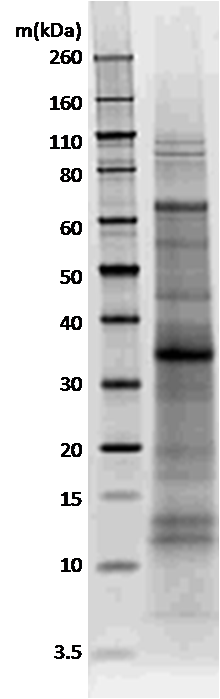


**Additional file 1: Fig. S2**

**
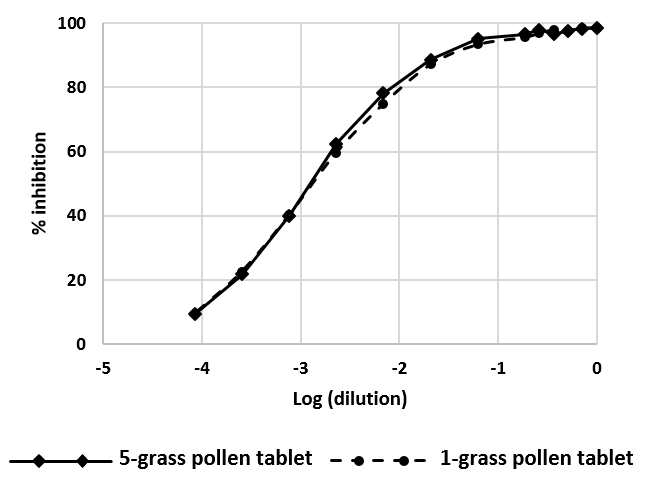
**

**Additional file 1: Fig. S3**

**
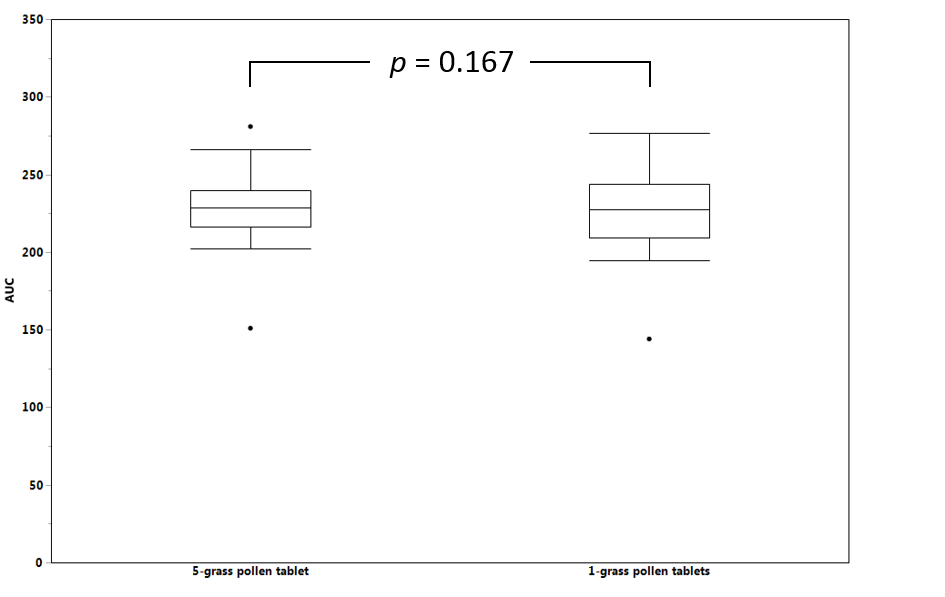
**

**Additional file 1: Fig. S4**

**
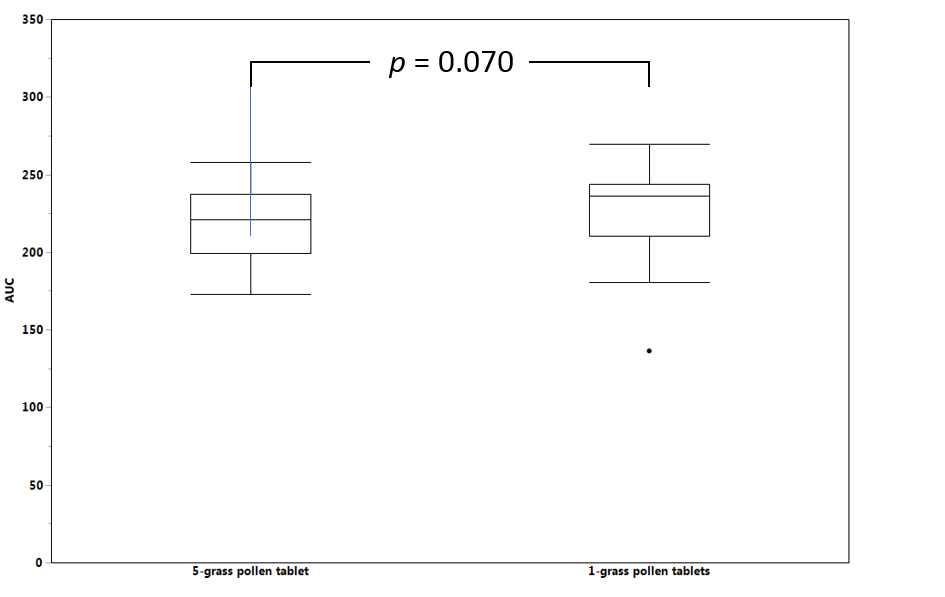
**

**Additional file 1: Fig. S5**

**
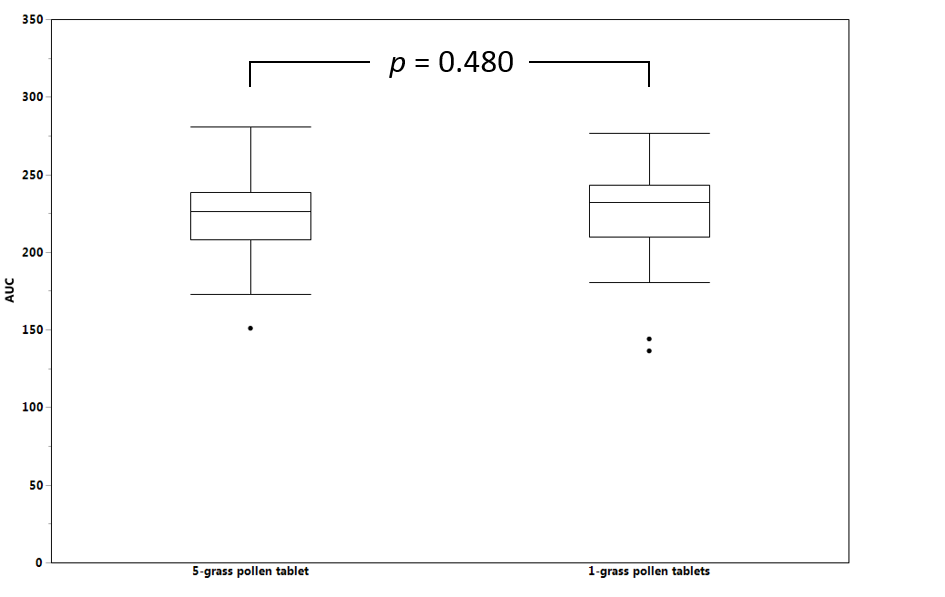
**

**Additional file 1: Fig. S6**

**
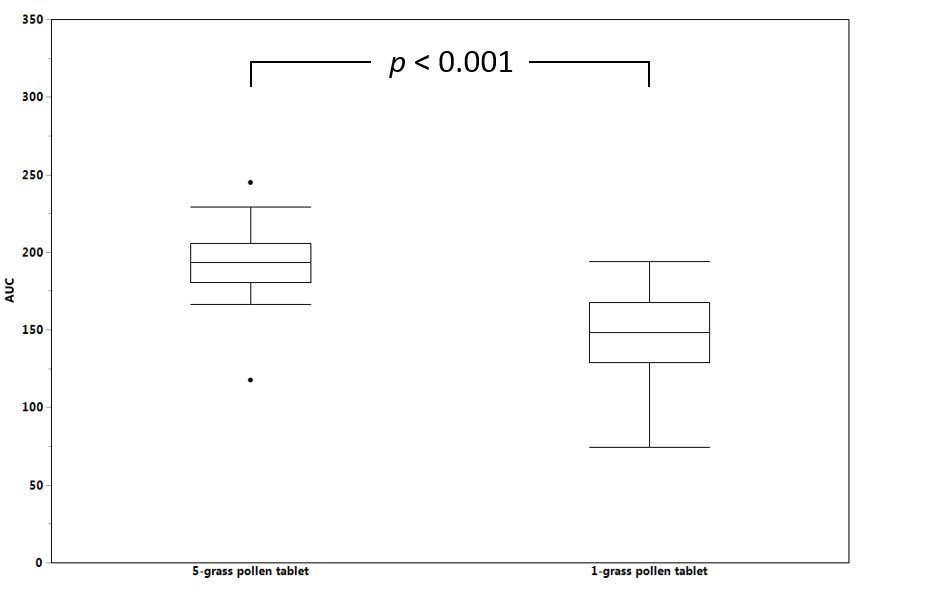
**

**Additional file 1: Fig. S7**

**
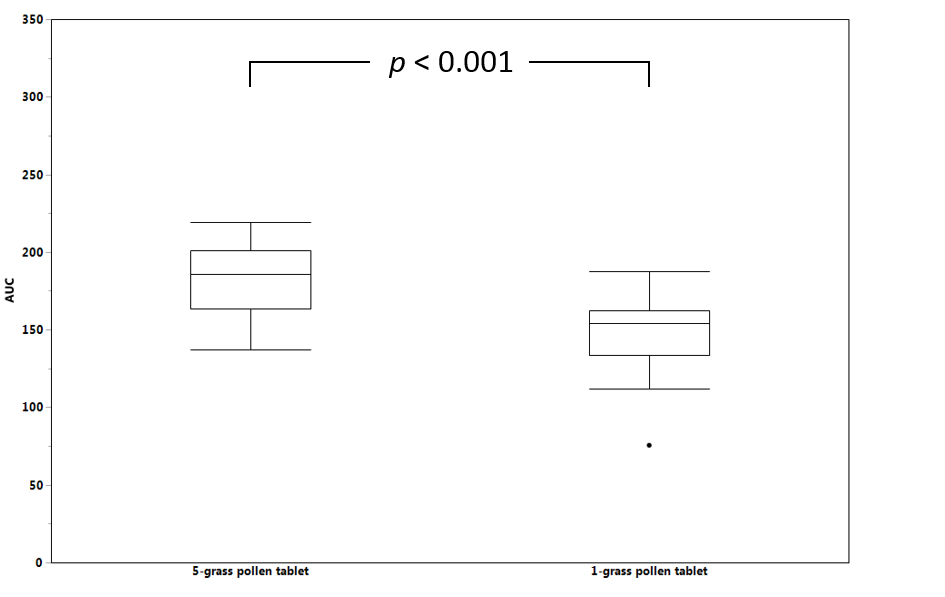
**

**Additional file 1: Fig. S8**

**
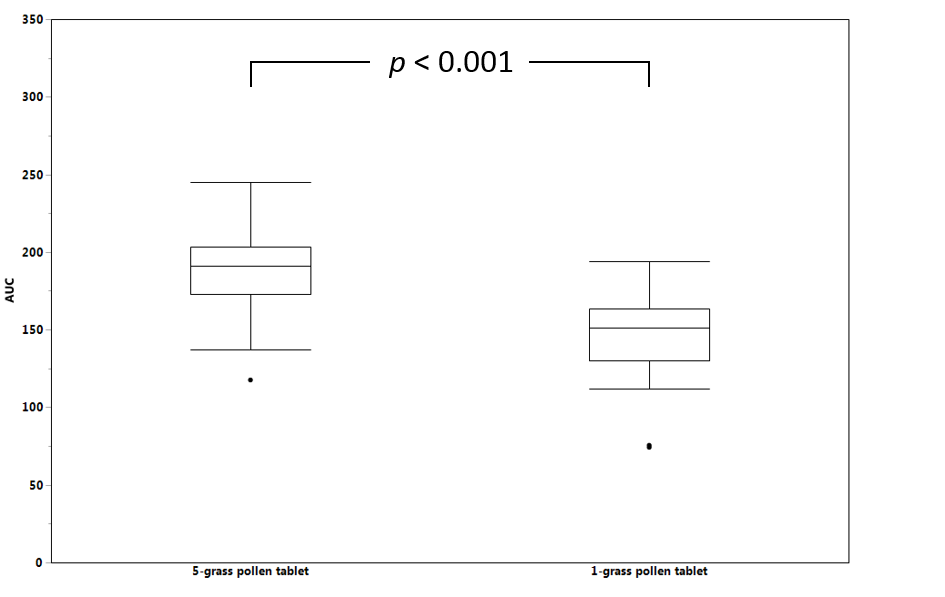
**

**Additional file 1: Table S1** Socio-demographic characteristics of the full analysis set of patients

|  |  |  |  |  |
| --- | --- | --- | --- | --- |
|  |  |  |  |  |
| Country |  | Spain | Sweden | Total |
|  |  |  |  |  |
|  |  |  |  |  |
| Number of patients |  | 20 | 22 | 42 |
|  |  |  |  |  |
|  |  |  |  |  |
| Age (years) | Mean (SD) | 35.7 (10.8) | 34.6 (9.6) | 35.1 (10.1) |
|  |  |  |  |  |
|  | RSD (%) | 30.4 | 27.7 | 28.8 |
|  |  |  |  |  |
|  | Median | 33.7 | 33.5 | 33.7 |
|  |  |  |  |  |
|  | Q1; Q3 | 25.1; 44.7 | 27.5; 43.0 | 26.5; 43.1 |
|  |  |  |  |  |
|  | Min; Max | 22.4; 54.7 | 19.0; 52.9 | 19.0; 54.7 |
|  |  |  |  |  |
|  |  |  |  |  |
| Gender | Male | 11 (55.0%) | 11 (50.0%) | 22 (52.4%) |
|  |  |  |  |  |
|  | Female | 9 (45.0%) | 11 (50.0%) | 20 (47.6%) |
|  |  |  |  |  |
|  |  |  |  |  |
| Number of years living in the area | Mean (SD) | 25.1 (13.5) | 32.4 (10.3) | 28.9 (12.4) |
|  |  |  |  |  |
|  | RSD (%) | 54.0 | 31.8 | 42.8 |
|  |  |  |  |  |
|  | Median | 23.5 | 31.5 | 29.5 |
|  |  |  |  |  |
|  | Q1; Q3 | 14.0; 34.0 | 26.0; 42.0 | 21.0; 39.0 |
|  |  |  |  |  |
|  | Min; Max | 6; 51 | 10; 52 | 6; 52 |
|  |  |  |  |  |
|  |  |  |  |  |

SD, standard deviation; RSD, relative standard deviation; Q1, lower quartile; Q3, upper quartile; Min, minimum; Max, maximum.

**Additional file 1: Table S2** Respiratory allergies (other than to grass pollens) of the full analysis set of patients

|  |  |  |  |  |
| --- | --- | --- | --- | --- |
|  |  |  |  |  |
| Country |  | Spain | Sweden | Total |
|  |  |  |  |  |
|  |  |  |  |  |
| Respiratory allergy | N | 20 | 22 | 42 |
|  |  |  |  |  |
|  | No | 3 (15.0%) | 5 (22.7%) | 8 (19.0%) |
|  |  |  |  |  |
|  | Yes | 17 (85.0%) | 17 (77.3%) | 34 (81.0%) |
|  |  |  |  |  |
|  | Missing | 0 | 0 | 0 |
|  |  |  |  |  |
|  |  |  |  |  |
| Causality | Alternaria | 1 (5.9%) | - | 1 (2.9%) |
|  |  |  |  |  |
|  | Birch pollen | - | 14 (82.4%) | 14 (41.2%) |
|  |  |  |  |  |
|  | Cat | 9 (52.9%) | 6 (35.3%) | 15 (44.1%) |
|  |  |  |  |  |
|  | Cypress pollen | 6 (35.3%) | - | 6 (17.6%) |
|  |  |  |  |  |
|  | Combination *D. pteronyssinus* and *D. farinae* | - | 2 (11.8%) | 2 (5.9%) |
|  |  |  |  |  |
|  | Dog | 4 (23.5%) | 6 (35.3%) | 10 (29.4%) |
|  |  |  |  |  |
|  | Olive tree pollen | 2 (11.8%) | - | 2 (5.9%) |
|  |  |  |  |  |
|  | Plane pollen | 4 (23.5%) | - | 4 (11.8%) |
|  |  |  |  |  |
|  | Other | 1 (5.9%) | 3 (17.6%) | 4 (11.8%) |
|  |  |  |  |  |
|  | Other combination | - | 1 (5.9%) | 1 (2.9%) |
|  |  |  |  |  |
|  |  |  |  |  |

N, number of patients for which the information was obtained.

**Additional file 1: Table S3** Food allergies of the full analysis set of patients

|  |  |  |  |  |
| --- | --- | --- | --- | --- |
|  |  |  |  |  |
| Country |  | Spain | Sweden | Total |
|  |  |  |  |  |
|  |  |  |  |  |
| Food allergy | N | 20 | 22 | 42 |
|  |  |  |  |  |
|  | No | 13 (65.0%) | 8 (36.4%) | 21 (50.0%) |
|  |  |  |  |  |
|  | Yes | 7 (35.0%) | 14 (63.6%) | 21 (50.0%) |
|  |  |  |  |  |
|  | Missing | 0 | 0 | 0 |
|  |  |  |  |  |
|  |  |  |  |  |
| Causality | Peanuts | - | 4 (28.6%) | 4 (19.0%) |
|  |  |  |  |  |
|  | Shellfish | 1 (14.3%) | 1 (7.1%) | 2 (9.5%) |
|  |  |  |  |  |
|  | Soy | - | 1 (7.1%) | 1 (4.8%) |
|  |  |  |  |  |
|  | Tree nuts | 3 (42.9%) | 1 (7.1%) | 4 (19.0%) |
|  |  |  |  |  |
|  | Other | 4 (57.1%) | 9 (64.3%) | 13 (61.9%) |
|  |  |  |  |  |
|  |  |  |  |  |

N, number of patients for which the information was obtained.

**Additional file 1: Table S4** Signs and symptoms of grass pollen allergies of the full analysis set of patients

|  |  |  |  |  |
| --- | --- | --- | --- | --- |
|  |  |  |  |  |
| Country |  | Spain | Sweden | Total |
|  |  |  |  |  |
|  |  |  |  |  |
| Rhinorrhoea | N | 20 | 22 | 42 |
|  |  |  |  |  |
|  | No | 2 (10.0%) | 1 (4.5%) | 3 (7.1%) |
|  |  |  |  |  |
|  | Yes | 18 (90.0%) | 21 (95.5%) | 39 (92.9%) |
|  |  |  |  |  |
|  | Missing | 0 | 0 | 0 |
|  |  |  |  |  |
|  |  |  |  |  |
| Wheezing | N | 20 | 22 | 42 |
|  |  |  |  |  |
|  | No | 9 (45.0%) | 9 (40.9%) | 18 (42.9%) |
|  |  |  |  |  |
|  | Yes | 11 (55.0%) | 13 (59.1%) | 24 (57.1%) |
|  |  |  |  |  |
|  | Missing | 0 | 0 | 0 |
|  |  |  |  |  |
|  |  |  |  |  |
| Nasal obstruction | N | 20 | 22 | 42 |
|  |  |  |  |  |
|  | No | 5 (25.0%) | 1 (4.5%) | 6 (14.3%) |
|  |  |  |  |  |
|  | Yes | 15 (75.0%) | 21 (95.5%) | 36 (85.7%) |
|  |  |  |  |  |
|  | Missing | 0 | 0 | 0 |
|  |  |  |  |  |
|  |  |  |  |  |
| Nasal itching | N | 20 | 22 | 42 |
|  |  |  |  |  |
|  | No | 3 (15.0%) | 2 (9.1%) | 5 (11.9%) |
|  |  |  |  |  |
|  | Yes | 17 (85.0%) | 20 (90.9%) | 37 (88.1%) |
|  |  |  |  |  |
|  | Missing | 0 | 0 | 0 |
|  |  |  |  |  |
|  |  |  |  |  |
| Sneezing | N | 20 | 22 | 42 |
|  |  |  |  |  |
|  | No | - | 4 (18.2%) | 4 (9.5%) |
|  |  |  |  |  |
|  | Yes | 20 (100.0%) | 18 (81.8%) | 38 (90.5%) |
|  |  |  |  |  |
|  | Missing | 0 | 0 | 0 |
|  |  |  |  |  |
|  |  |  |  |  |
| Ocular itching | N | 20 | 22 | 42 |
|  |  |  |  |  |
|  | No | 3 (15.0%) | - | 3 (7.1%) |
|  |  |  |  |  |
|  | Yes | 17 (85.0%) | 22 (100.0%) | 39 (92.9%) |
|  |  |  |  |  |
|  | Missing | 0 | 0 | 0 |
|  |  |  |  |  |
|  |  |  |  |  |
| Eyelid swelling | N | 20 | 22 | 42 |
|  |  |  |  |  |
|  | No | 9 (45.0%) | 6 (27.3%) | 15 (35.7%) |
|  |  |  |  |  |
|  | Yes | 11 (55.0%) | 16 (72.7%) | 27 (64.3%) |
|  |  |  |  |  |
|  | Missing | 0 | 0 | 0 |
|  |  |  |  |  |
|  |  |  |  |  |
| Tearing | N | 20 | 22 | 42 |
|  |  |  |  |  |
|  | No | 11 (55.0%) | 4 (18.2%) | 15 (35.7%) |
|  |  |  |  |  |
|  | Yes | 9 (45.0%) | 18 (81.8%) | 27 (64.3%) |
|  |  |  |  |  |
|  | Missing | 0 | 0 | 0 |
|  |  |  |  |  |
|  |  |  |  |  |
| Other | N | 4 | 7 | 11 |
|  |  |  |  |  |
|  | Yes | 4 (100.0%) | 7 (100.0%) | 11 (100.0%) |
|  |  |  |  |  |
|  | Missing | 16 | 15 | 31 |
|  |  |  |  |  |
|  |  |  |  |  |

N, number of patients for which the information was obtained.

**Additional file 1: Table S5** Diagnosis of grass pollen allergy of the full analysis set of patients

|  |  |  |  |  |  |
| --- | --- | --- | --- | --- | --- |
|  |  |  |  |  |  |
| Country |  | Spain | Sweden | Total |  |
|  |  |  |  |  |  |
|  |  |  |  |  |  |
| Time from first symptoms of grass-pollen (years) | N | 20 | 22 | 42 |  |
|  |  |  |  |  |  |
|  | Missing | 0 | 0 | 0 |  |
|  |  |  |  |  |  |
|  | Mean (SD) | 16.0 (10.0) | 18.8 (10.8) | 17.5 (10.4) |  |
|  |  |  |  |  |  |
|  | RSD (%) | 62.4 | 57.5 | 59.5 |  |
|  |  |  |  |  |  |
|  | Median | 14.0 | 16.0 | 15.1 |  |
|  |  |  |  |  |  |
|  | Q1; Q3 | 10.0; 21.9 | 10.2; 28.8 | 10.0; 25.7 |  |
|  |  |  |  |  |  |
|  | Min; Max | 3.05; 39.9 | 5.9; 42.7 | 3.05; 42.7 |  |
|  |  |  |  |  |  |
|  |  |  |  |  |  |
| Duration of grass pollen allergy (years) | N | 20 | 20 | 40 |  |
|  |  |  |  |  |  |
|  | Missing | 0 | 2 | 2 |  |
|  |  |  |  |  |  |
|  | Mean (SD) | 12.2 (11.2) | 17.0 (12.9) | 14.6 (12.2) |  |
|  |  |  |  |  |  |
|  | RSD (%) | 92.1 | 76.2 | 83.6 |  |
|  |  |  |  |  |  |
|  | Median | 11.3 | 10.4 | 10.7 |  |
|  |  |  |  |  |  |
|  | Q1; Q3 | 1.4; 20.7 | 7.3; 29.2 | 4.3; 22.9 |  |
|  |  |  |  |  |  |
|  | Min; Max | -.06; 35.7 | 0; 42.7 | -.06; 42.7 |  |
|  |  |  |  |  |  |
|  |  |  |  |  |  |
| Average duration of symptoms of allergic rhinoconjunctivitis (weeks) | N | 20 | 22 | 42 |  |
|  |  |  |  |  |  |
|  | Missing | 0 | 0 | 0 |  |
|  |  |  |  |  |  |
|  | Mean (SD) | 7.2 (3.3) | 13.3 (3.3) | 10.4 (4.5) |  |
|  |  |  |  |  |  |
|  | CV (%) | 45.7 | 25.1 | 43.5 |  |
|  |  |  |  |  |  |
|  | Median | 6.0 | 12.0 | 11.0 |  |
|  |  |  |  |  |  |
|  | Q1; Q3 | 5.5; 9.5 | 12.0; 16.0 | 6.0; 14.0 |  |
|  |  |  |  |  |  |
|  | Min; Max | 2; 16 | 4; 20 | 2; 20 |  |
|  |  |  |  |  |  |
|  |  |  |  |  |  |
| Symptomatic treatment | N | 20 | 22 | 42 |  |
|  |  |  |  |  |  |
|  | Missing | 0 | 0 | 0 |  |
|  |  |  |  |  |  |
|  | No | 4 (20.0%) | - | 4 (9.5%) |  |
|  |  |  |  |  |  |
|  | Yes | 16 (80.0%) | 22 (100.0%) | 38 (90.5%) |  |
|  |  |  |  |  |  |
|  |  |  |  |  |  |
| AIT | N | 20 | 22 | 42 |  |
|  |  |  |  |  |  |
|  | Missing | 0 | 0 | 0 |  |
|  |  |  |  |  |  |
|  | No | 16 (80.0%) | 20 (90.9%) | 36 (85.7%) |  |
|  |  |  |  |  |  |
|  | Yes | 4 (20.0%) | 2 (9.1%) | 6 (14.3%) |  |
|  |  |  |  |  |  |
|  |  |  |  |  |  |

N, number of patients for which the information was obtained; SD, standard deviation; RSD, relative standard deviation; Q1, lower quartile; Q3, upper quartile; Min, minimum; Max, maximum.

**Additional file 1: Table S6** Timothy pollen-specific IgE testing of the full analysis set of patients

|  |  |  |  |
| --- | --- | --- | --- |
|  |  |  |  |
| Country | Spain | Sweden | Total |
|  |  |  |  |
|  |  |  |  |
| N | 15 | 21 | 36 |
|  |  |  |  |
| Missing | 5 | 1 | 6 |
|  |  |  |  |
| Mean (kU/L) (SD) | 40.8 (27.2) | 24.1 (24.0) | 31.1 (26.3) |
|  |  |  |  |
| RSD (%) | 66.5 | 99.5 | 84.8 |
|  |  |  |  |
| Median | 29.5 | 17.0 | 23.9 |
|  |  |  |  |
| Q1; Q3 | 21.0; 67.1 | 8.2; 34.0 | 9.7; 40.8 |
|  |  |  |  |
| Min; Max | 12.4; 100 | 1.4; 91 | 1.4; 100 |
|  |  |  |  |
|  |  |  |  |

N, number of patients for which the information was obtained; SD, standard deviation; RSD, relative standard deviation; Q1, lower quartile; Q3, upper quartile; Min, minimum; Max, maximum.

**Additional file 1: Table S7** Skin prick testing to grass pollen of the full analysis set of patients

|  |  |  |  |  |
| --- | --- | --- | --- | --- |
|  |  |  |  |  |
| Country |  | Spain | Sweden | Total |
|  |  |  |  |  |
|  |  |  |  |  |
| Wheal diameter for grass pollen (mm) | N | 17 | 22 | 39 |
|  |  |  |  |  |
|  | Missing | 3 | 0 | 3 |
|  |  |  |  |  |
|  | Mean (SD) | 8.3 (2.7) | 11.0 (7.3) | 9.8 (5.9) |
|  |  |  |  |  |
|  | RSD (%) | 32.1 | 66.4 | 59.6 |
|  |  |  |  |  |
|  | Median | 8.0 | 8.0 | 8.0 |
|  |  |  |  |  |
|  | Q1; Q3 | 6.0; 10.0 | 6.0; 16.0 | 6.0; 12.0 |
|  |  |  |  |  |
|  | Min; Max | 4; 13 | 2; 26 | 2; 26 |
|  |  |  |  |  |
|  |  |  |  |  |
| Wheal diameter for negative control (mm) | N | 17 | 22 | 39 |
|  |  |  |  |  |
|  | Missing | 3 | 0 | 3 |
|  |  |  |  |  |
|  | Mean (SD) | 0.0 (0.0) | 0.0 (0.0) | 0.0 (0.0) |
|  |  |  |  |  |
|  | RSD (%) | N/A | N/A | N/A |
|  |  |  |  |  |
|  | Median | 0.0 | 0.0 | 0.0 |
|  |  |  |  |  |
|  | Q1; Q3 | 0.0; 0.0 | 0.0; 0.0 | 0.0; 0.0 |
|  |  |  |  |  |
|  | Min; Max | 0; 0 | 0; 0 | 0; 0 |
|  |  |  |  |  |
|  |  |  |  |  |
| Wheal diameter for positive control (mm) | N | 17 | 22 | 39 |
|  |  |  |  |  |
|  | Missing | 3 | 0 | 3 |
|  |  |  |  |  |
|  | Mean (SD) | 4.9 (1.3) | 5.3 (1.3) | 5.2 (1.3) |
|  |  |  |  |  |
|  | RSD (%) | 26.3 | 24.9 | 25.4 |
|  |  |  |  |  |
|  | Median | 5.0 | 5.0 | 5.0 |
|  |  |  |  |  |
|  | Q1; Q3 | 4.0; 5.0 | 4.0; 6.0 | 4.0; 6.0 |
|  |  |  |  |  |
|  | Min; Max | 3; 9 | 3; 9 | 3; 9 |
|  |  |  |  |  |
|  |  |  |  |  |
| Skin prick test result for grass pollen | N | 17 | 22 | 39 |
|  |  |  |  |  |
|  | Positive | 17 (100.0%)* | 22 (100.0%) | 39 (100.0%)* |
|  |  |  |  |  |
|  | Missing | 3 | 0 | 3 |
|  |  |  |  |  |
|  |  |  |  |  |

N, number of patients for which the information was obtained; SD, standard deviation; RSD, relative standard deviation; Q1, lower quartile; Q3, upper quartile; Min, minimum; Max, maximum; N/A, not applicable. *Spanish patient #00105 turned out to be erroneously considered skin prick test positive and had no grass pollen-specific IgE test performed; it was therefore excluded from the per protocol set.
